# How Do Anti-SARS-CoV-2 mRNA Vaccines Protect from Severe Disease?

**DOI:** 10.3390/ijms231810374

**Published:** 2022-09-08

**Authors:** Maurizio Federico

**Affiliations:** National Center for Global Health, Istituto Superiore di Sanità, Viale Regina Elena, 299, 00161 Rome, Italy; maurizio.federico@iss.it; Tel.: +39-06-4990-6016

**Keywords:** SARS-CoV-2, mRNA vaccines, alveolar macrophages, TGF-β

## Abstract

COVID-19 pathogenesis develops in two phases. First, Severe Acute Respiratory Syndrome Coronavirus (SARS-CoV)-2 spreads within the epithelial cells of the mucosa of upper and, possibly, lower respiratory tracts. While the virus dissemination can be controlled by an emerging adaptive host immune response, if the virus diffuses to the pulmonary alveoli, a potentially lethal mechanism can arise in the second phase. It consists of an uncontrolled burst of cytokines/inflammatory factors (i.e., cytokine storm), leading to the insurgence of respiratory symptoms and, consequently, multi-organ failures. Messenger (m)RNA-based vaccines represent the most innovative approach in terms of prophylaxis against SARS-CoV-2-induced disease. The cumulating data indicate that the response to mRNA vaccines is basically ineffective to counteract the viral replication in the upper respiratory tracts, while showing efficacy in containing the development of severe disease. Considering that the antiviral immunity elicited by intramuscularly delivered mRNA vaccines is expected to show similar quantitative and qualitative features in upper and lower respiratory tracts, the different outcomes appear surprising and deserve accurate consideration. In this review, a still unexplored mechanism accounting for the mRNA vaccine effect against severe disease is proposed. Based on well-established experimental evidence, a possible inhibitory effect on alveolar macrophages as a consequence of the diffusion of the extracellular and/or cell-associated Spike protein can be envisioned as a key event counteracting the cytokine storm. This benefit, however, may be associated with defects in the immune functions of macrophages in other tissues whose possible consequences deserve careful evaluation.

## 1. Introduction

In December 2019, the Wuhan Municipal Health Commission (China) reported to the World Health Organization (WHO) a cluster of pneumonia cases of unknown etiology in the city of Wuhan in the Chinese province of Hubei. In March 2020, the WHO declared a pandemic status. The spread of Severe Acute Respiratory Syndrome Coronavirus (SARS-CoV)-2 paralleled with the race for possible countermeasures. Several vaccines were developed and distributed within an unprecedented short time. They included, considering those with a global degree of spread only, mRNA vaccines (Pfizer/BioNTech, New York, NY, USA; Moderna, Cambridge, MA, USA), adenoviral vector-based vaccines (Sputnik V, R-Farm, Moscow, Russia; Covishield, Oxford/AstraZeneca formulation, and AZD 1222, Oxford/AstraZeneca, Cambridge, UK; Johnson & Johnson, Janssen Pharmaceuticals, Beerse, Belgium), inactivated vaccines (Sinovac, Bejing, China; Sinopharm, Bejing, China), and protein vaccines (Nuvaxovid, NovaVax, Gaithersburg, MD, USA).

All of these vaccine preparations were designed to elicit anti-Spike protein immune responses, and for the most part, their effectiveness was expected to rely on the induction of neutralizing antibodies. After the early period, when both AstraZeneca and Johnson & Johnson vaccines represented diffuse options, mRNA-based vaccines became the vaccines almost exclusively distributed in Western countries.

The mRNA vaccines are composed of in vitro synthesized mRNA molecules coding for full-length SARS-CoV-2 Spike glycoproteins from the ancestral strain (i.e., Wuhan isolate) in a prefusion conformation. Upon injection in the deltoid muscle, mRNA molecules enter the cells by virtue of their encapsulation into synthetic lipid nanovesicles. The mRNA-targeted cells express the Spike protein in trimeric complexes on their plasma membrane in a way that it can be sensed by the immune system, which reacts by generating anti-Spike protein antibodies.

The second injection of anti-SARS-CoV-2 mRNA vaccines leads to the production of extraordinarily high levels of anti-Spike protein antibodies as measurable in serum [1,2]. At the peak of antibody production, Spike protein-binding antibodies can also be detected at the viral port of entry, i.e., the mucosa of the upper respiratory tract [3,4]. Regrettably, however, it is not associated with a significant virus neutralization and reduction in virus replication [5,6]. This unfortunate effect was first documented for both the Alpha and Delta variants and is now even more evident with the currently widespread Omicron variant [7,8]. Despite the lack of efficacy in blocking virus transmission, the mRNA vaccines demonstrated a measurable efficacy in controlling severe forms of COVID-19, most frequently observed with the Alpha and Delta variants [9,10]. This effect led to reduced hospitalizations, ICU accessions, and deaths.

Such a dichotomy appears to have a non-obvious explanation. In fact, the vaccine-induced immune stimulus is provided peripherally by the intramuscular injection. Hence, the quality and extent of the vaccine-induced anti-Spike protein immunity responses are expected to be similar in the upper and lower respiratory tracts [11,12].

In this review, possible mechanisms accounting for the dichotomous effects of mRNA-based anti-SARS-CoV-2 vaccines are discussed. It is conceivable that the control over severe COVID-19 would be the consequence of a skewing of functions of specific types of immune cells at the pulmonary level, most likely the alveolar macrophages.

## 2. The Biphasic COVID-19 Pathogenesis

**Figure 1:**
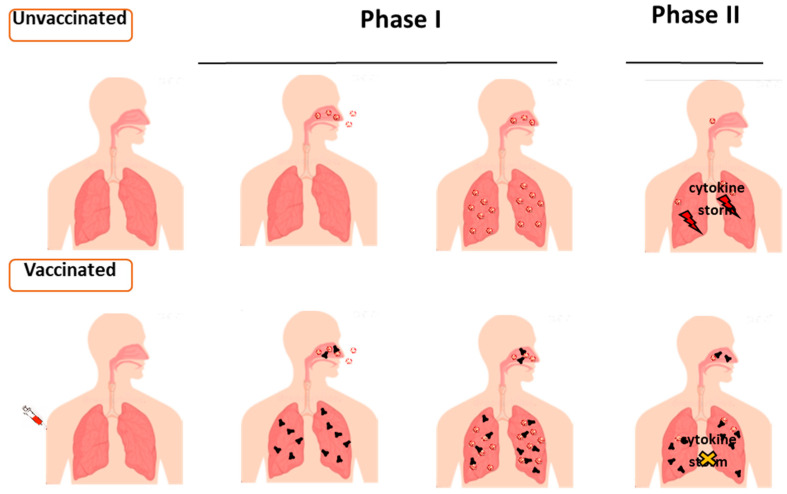
COVID-19 progression in unvaccinated and vaccinated subjects. COVID-19 recognizes two distinct pathogenetic phases, where SARS-CoV-2 spread into airways drives the first one. The second phase, culminating in the intrapulmonary cytokine storm, can be counteracted by the effects of vaccination.

The course of COVID-19 flows into two functionally correlated but distinct phases [13]. The first one is governed by the viral diffusion in airways, whereas in the second phase, the pathogenetic effects are driven by inflammatory factors released as the indirect consequence of the ongoing viral spread (Figure 1).

In detail, in the first instance, the infecting virus replicates mainly in the angiotensin-converting enzyme (ACE)-2/transmembrane serine protease (TMPRSS)-2-expressing ciliated epithelial cells of the nasal mucosa [14]. Thereafter, the virus can spread into the lower respiratory tract reaching, in most severe cases, alveolar cells. In the bronchial epithelium, the virus attacks ciliated cells, mucus-secreting cells, and club cells. The virus-replicating cells are killed, leading to mucosal epithelium damage, which can be eventually repaired by the replication of basal stem-cells. The virus-infected ciliated cells from post-mortem lung samples expressed CCL2, CCL8, and CCL11 chemokines in the absence of type I and III IFNs [15]. When the virus spreads to the alveoli, alveolar type II epithelial cells become readily infected, thereby releasing type I and III interferons as well as chemokines, which, in turn, attract and activate the immune cells, including macrophages and lymphocytes. In this way, a potent, self-propagating inflammatory process initiates, leading to severe damage of the gas exchange units. The alveolar macrophages, which do not replicate SARS-CoV-2 [16], are the key players in the inflammatory process. It takes place in both the alveolar lumen and basolateral side, thereby generating hypoxemia, rapidly leading to the COVID-19-related acute respiratory distress syndrome (ARDS). ARDS occurs when fluid builds up in the alveoli. The fluid keeps lungs from filling with enough air, thereby depriving organs of the needed oxygen. ARDS is the most serious complication of a SARS-CoV-2 infection. It is a consequence of the cytokine storm leading to respiratory epithelium damage [17], with both macrophages and lymphocytes secreting high levels of inflammatory cytokines, e.g., IL-6, GM-CSF [18].

In this scenario, the inoculation of mRNA vaccines has scarce/no effects on virus replication in the upper respiratory tract, but easily recognizable protective effects against the cytokine storm-driven severe disease. A detailed description of the antiviral immunity induced in both the upper and lower respiratory tracts compared to that generated in the peripheral circulation would help to gain insights into the actual immunologic effects induced by the mRNA vaccines.

## 3. The Immunity Induced by mRNA Vaccines in Blood and Respiratory Tracts

The immune response to anti-SARS-CoV-2 vaccines is commonly measured in the serum and circulatory cells. The analysis in the serum of subjects injected with current mRNA-based anti-SARS-CoV-2 vaccines reproducibly demonstrated the generation of a robust response in terms of induction of anti-Spike protein antibodies. The virus-neutralizing antibody sub-families include those specific to the receptor-binding domain (RBD), and the anti-N-terminal domain (NTD). IgGs are the predominant class of anti-Spike protein antibodies in the serum of vaccinees showing, unfortunately, a limited half-life [19]. In addition to antibodies, the mRNA vaccines induce circulatory anti-Spike protein memory B cells (MBCs) [20,21,22].

However, the battle against the virus begins in the upper respiratory tracts and can progress to the pulmonary alveoli. Hence, an efficient antiviral adaptive immunity should be most appropriately elicited within the respiratory tracts, where the immune system is highly specialized and compartmentalized [23]. This applies to humoral immunity, considering that the most efficient neutralizing antibody class in the airway mucosa are dimeric IgAs [24], which are virtually absent in the serum, as well as to cellular immunity, which develops autonomously from the events occurring in both the peripheral circulation and lymphoid organs. Their continuous loss through intraepithelial migration towards the airways is constantly replenished by homeostatic proliferation. Resident memory B cells (BRMCs) play a key role in the duration of humoral antiviral immunity in the lungs [25].

Concerning the immunity induced by mRNA vaccines in the upper respiratory ways, the seminal studies carried out by Planas et al. in humans demonstrated the absence of neutralization activity in nasal swabs after 2 weeks from the second injection of an mRNA vaccine. Conversely, high levels of binding activity (presumably due to non-neutralizing anti-Spike protein IgGs) were found in the presence of high titers of both binding and neutralizing antibodies in the serum [3]. More recent works have essentially confirmed these data [4,26,27]. As a direct consequence, the levels of viral replication in oral mucosa of vaccinated and unvaccinated subjects scored similarly [5,6].

On the other hand, reliable data on anti-Spike protein immunity in the lungs of vaccinated human subjects were very recently provided by Tang et al., who analyzed both neutralization activity and cell immunity in bronchoalveolar lavage fluids (BALFs) from vaccinated compared to unvaccinated subjects [28]. They found that the neutralization activity in the BALFs from vaccinated subjects scored just above the sensitivity threshold with the Delta variant and was undetectable with the Omicron BA1.1 variant. On the other hand, RBD-specific B cells were found in BALFs of only 21% of vaccinated subjects, in the total absence, however, of Spike-protein-specific CD4^+^ and CD8^+^ T lymphocytes. Conversely, both neutralization activity and easily detectable Spike protein specific cell immunity were observed in the BALFs from unvaccinated/convalescent subjects.

Therefore, in humans, despite anti-SARS-CoV-2 mRNA vaccines inducing high levels of anti-Spike protein circulating antibodies, both the antiviral neutralizing activity and cellular immunity were either severely limited or absent in the respiratory tracts.

## 4. Neglected Aspects of mRNA Vaccine Pharmacodynamics

The pharmacodynamics of the current anti-SARS-CoV-2 mRNA vaccines, i.e., how they influence the host physiology, was rarely considered in both pre-clinic and clinic studies. However, two seminal papers help to shed light regarding the fate of the vaccine mRNA molecules.

The first one describes accurate studies carried out in vaccinated cynomolgus macaques [29]. It clearly demonstrated that, as soon as 4 h after vaccine injection, mRNA molecules are internalized by the immune cells at both the site of injection and proximal lymph nodes in amounts appearing inversely proportional to the distance from the point of injection. The monocytes were found to be the immune cells most efficiently internalizing vaccine mRNA in both the muscle tissues and lymph nodes, where up to 80% of the infiltrated monocytes scored positive for the presence of vaccine mRNA as early as 16 h after injection. Vaccine mRNA was also internalized by 40 to 80% of the infiltrating CD44-positive B and T lymphocytes, as well as by similar percentages of the infiltrating dendritic cells. A direct consequence of mRNA internalization in long-lived circulating immune cells would be that the Spike protein can be persistently expressed in several distal tissues. In such a context, both high local concentrations of the Spike protein and the membrane-associated Spike protein can interact with the cells expressing the ACE-2 receptor, thereby influencing their physiology and functions.

On the other hand, very recently published data obtained in humans demonstrated that both the vaccine mRNA and Spike protein persist for many weeks in lymph nodal germinal centers [30]. Through elegant results obtained by in situ hybridization and immunohistochemical analyses, the presence of both the mRNA and Spike protein were documented in ipsilateral axillary core lymph node biopsies. Quite interestingly, more than 50,000 probe spots/mm^2^ of lymph node tissue were found 37 days after the second dose of the BNT162b2 vaccine, and about 1000 probe spots/mm^2^ lasted at day 60 after injection. At these times, the authors reported the presence of “abundant” cell-free Spike antigens in the same tissue specimens.

Based on the data from these two studies, one can conclude that, although the injected mRNA vaccine is expected to be internalized by the muscle cells, part of the inoculum diffuses and circulates, thereby being taken up by the immune cells infiltrating the muscle tissue as well as the lymph node cells. When the vaccine mRNA enters long-lasting, circulating cells (i.e., monocytes, lymphocytes), both cell-associated and cell-free Spike protein can diffuse towards the lungs, as well as the other peripheral tissues.

The consequences of these findings would be manifold and, at least in part, not easy to predict.

## 5. Intracellular Signaling Induced by the Spike-Protein-Induced ACE-2 Engagement

The SARS-CoV-2 Spike protein binds the ACE-2 cell membrane receptor, which acts as a major cell factor in the virus entry process. ACE-2 is a key regulator in the renin–angiotensin–aldosterone system involved in the control of blood pressure. ACE-2 catalyzes the conversion of angiotensin I, a decapeptide, to angiotensin 1–9, which may be converted to smaller, vasodilator angiotensin peptides (e.g., angiotensin 1–7) by ACE in the lungs. ACE-2 also binds angiotensin II, i.e., an octapeptide generated by the ACE-driven cleavage of angiotensin I, to produce angiotensin 1–7. Through their different angiotensin peptide products, ACE and ACE-2 exert different effects on blood pressure regulation and inflammation [31,32]. The interaction between ACE-2 and angiotensin II switches various signaling pathways, including serine/threonine kinases, ERK, JNK/MAPK, and PKC [33]. Thereby, angiotensin II induces the release of TGF-β, IL-6, and TNF-α also through G protein-coupled receptor activation and interaction with mineralocorticoid receptors [34,35,36,37,38]. The effects of the interaction between the Spike protein and ACE-2 basically recapitulate those described for the interaction with its natural ligand [39]. Moreover, the ACE-2 downregulation following the binding with the Spike protein can lead to an increase in local blood pressure, since the decrease in ACE-2 results in a lower conversion of angiotensin I/II to the vasodilator angiotensin 1–7 [32,40]. This effect leads to a local increase in vascular permeability, inflammation, and coagulation. In addition, Spike-protein-induced ACE-2 downregulation in the vascular endothelial cells generates a block of mitochondrial functions [41]. In these cells, the Spike protein can also induce integrin α5β1-dependent signaling leading to nuclear translocation of NF-κB, with consequent expression of the leukocyte adhesion molecules VCAM-1 and ICAM-1; coagulation factors; and the release of TNFα, IL-1β, and IL-6 pro-inflammatory cytokines [42]. Similar activation mechanisms were described in both macrophages and dendritic cells [43,44,45].

In summary, the literature data consistently support the idea that the immediate effect of the ACE-2/Spike protein interaction is the release of soluble factors, whose downstream consequences depend on the target cell type.

## 6. How mRNA Vaccines Can Control the SARS-CoV-2-Induced Cytokine Storm

Sadarangani et al. proposed the idea that anti-SARS-CoV-2 vaccines may protect from severe disease through an antibody-independent action [46]. The mRNA vaccine delivery leads to the expression of both free and cell-associated Spike proteins, whose interaction with ACE-2 can induce inflammation. On the other hand, these vaccines render the host resistant to the SARS-CoV-2-induced inflammatory cascade through a mechanism seemingly independent from the elicited immune response. How can this apparently conflicting evidence be reconciled?

The alveolar macrophages are the key players in the SARS-CoV-2-induced cytokine storm. They express high levels of ACE-2 [47], meanwhile resisting the SARS-CoV-2 replication [16,48]. The persistence of mRNA vaccine-related effectors [32], possibly including anti-idiotype antibodies against anti-Spike protein antibodies [49], could account for a protracted stimulation of alveolar macrophages and, consequently, their desensitization, similar to what was recently described in a model of simian immunodeficiency virus chronic infection [50].

**Figure 2:**
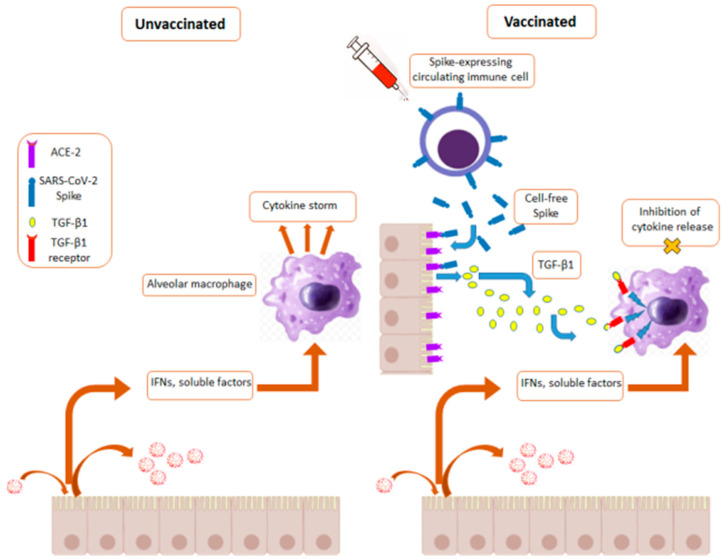
A possible mechanism underlying the vaccine-induced anti-inflammatory action. Both the free and cell-associated Spike proteins can interact with the alveolar epithelial cells, which thereby release TGF-β1. This stimulus induces a state of unresponsiveness in bystander alveolar macrophages. As a result, in cases where SARS-CoV-2 spreads into the pulmonary alveoli, the virus-induced cytokine storm can be severely impaired.

How could the process of widespread desensitization of the alveolar macrophages occur in vaccinated individuals? It is known that the cells interacting with the Spike protein can be induced to release several types of soluble factors, including type I IFN, IL-21 (i.e., a cytokine regulating both the NK cells and CD8^+^ T lymphocytes), IL-4, and the metalloprotease MMP-9. In addition, it was reported that the Spike protein induces the release of TGF-β1 from different cell types, including the pulmonary epithelial cells, the endothelial cells, and B-lymphocytes [51,52,53]. TGF-β molecules play a key role in the homeostasis of alveolar macrophages [54]. In addition, the stimulation of the TGF-β membrane receptor in alveolar macrophages leads to the release of TGF-β molecules through a type of self-amplification mechanism [54]. Most importantly, it was recently reported that the treatment of alveolar macrophages with TGF-β1 strongly inhibits the intracellular pro-inflammatory signaling, in particular, in those dependent on type I IFN [55]. On this basis, it is tempting to hypothesize that, in vaccinated individuals, alveolar macrophages interacting with Spike-protein-induced TGF-β1 become unresponsive to inflammatory stimuli, meanwhile being induced to release TGF-β1, whose diffusion can contribute to the extended unresponsiveness of bystander alveolar macrophages. In this context, the soluble factors released by the SARS-CoV-2-infected alveolar type II epithelial cells of vaccinees failed to switch on the inflammatory amplification typically generated by alveolar macrophages in COVID-19 pathogenesis. This condition can lead to a block of the life-threatening cytokine storm so that the progression towards severe disease and death is hampered (Figure 2), while the virus can be cleared by the naturally induced adaptive immune response.

**Figure 3:**
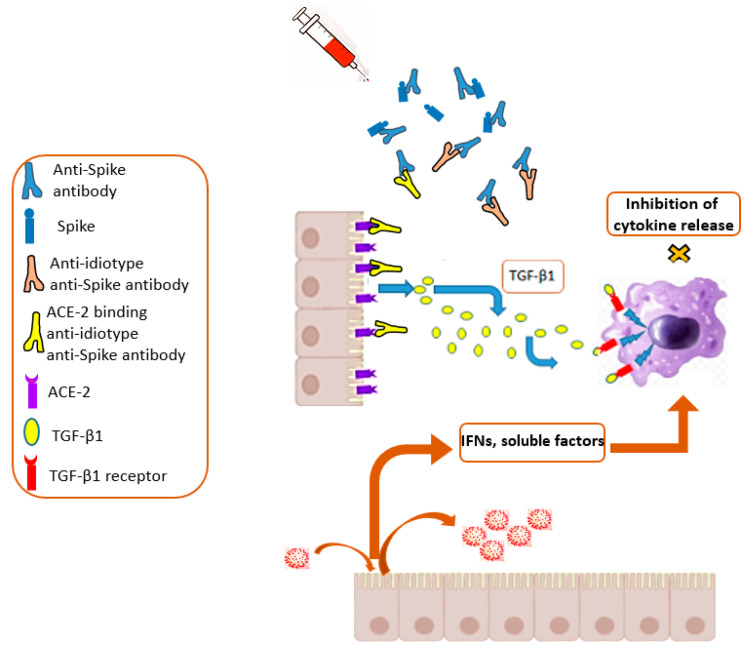
The anti-inflammatory action of anti-idiotype anti-Spike protein antibodies. The quite abundant production of anti-Spike protein antibodies generated after vaccination can elicit an antibody response against their variable domains. Part of these anti-idiotype anti-Spike protein antibodies (in yellow) can bind ACE-2 by virtue of a mechanism of molecular mimicry. Similar to what is possibly occurring with the Spike protein, this interaction at the level of alveolar epithelial cells can result in a release of TGF-β1, whose ultimate effect might be a functional unresponsiveness of alveolar macrophages. When this phenomenon occurs in other districts, immune defects with unpredictable consequences could be generated.

It is noteworthy that the potency and persistence of the humoral immune response induced by the current mRNA vaccines are optimal pre-requisites for the production of anti-idiotype anti-Spike protein antibodies [49]. Through the phenomenon of molecular mimicry, the sub-families of these antibodies could bind and activate ACE-2 receptors, leading to effects superimposable on those induced by the Spike protein (Figure 3). The most alarming consequence would be the induction of a sort of anti-ACE-2 autoimmunity, which could persist indefinitely.

## 7. Conclusions

The fact that the mRNA-based anti-SARS-CoV-2 vaccines protect from severe disease while being ineffective in the mucosa of upper respiratory tracts can be hardly explained in terms of adaptive immunity only. The involvement of both B- and T-cell immunity was called into question to explain the vaccine-induced protection. However, anti-Spike protein cell immunity seems basically absent in the lungs of vaccinees [28].

After infection, the replication in the upper and then the lower respiratory tracts of SARS-CoV-2 in immunocompetent hosts is accompanied by a vigorous natural antiviral immune response, which in most cases is sufficient to limit the viral spread in the lungs. Either transient or, as seen in the elderly, chronic immune defects can allow the virus to replicate in the lung tissues more extensively, with the switching on of the alveolar macrophage-mediated, life-threatening cytokine storm. This phenomenon, typically leading to ARDS, could be effectively controlled by the Spike-protein-dependent inhibitory effect induced by the vaccines in the alveolar macrophages

This hypothesis does not necessarily exclude a contribution of vaccine-induced neutralizing antibodies to the control of the disease. However, it remains unclear how the vaccine-induced antibody-dependent neutralization would act exclusively at the level of the pulmonary alveoli. An idea concerning a recruitment in the infected lungs of vaccine-induced, circulating Spike-protein-specific B- and T-lymphocytes was invoked to explain why the protection from severe disease is not supported by the clinical data [28]. In any case, this hypothesis appears unlikely since a similar mechanism would occur in the upper respiratory tracts as well, where, conversely, the virus replicates efficiently despite vaccination. On this subject, significant notions come from immunology studies carried out with vaccines against other respiratory viruses. In particular, the intramuscular injection of an anti-Respiratory Syncytial Virus vaccine elicited immunities in both the nose and lungs showing identical anti-viral efficacies [11]. In addition, the intramuscular injection of an adenoviral vector-based anti-flu vaccine induced quite balanced humoral and cellular anti-viral immune responses in the upper and lower respiratory tracts [12]. Hence, it remains unclear why, on the contrary, anti-SARS-CoV-2 vaccines would induce such unbalanced antiviral immune responses in different respiratory districts.

The mRNA vaccine-dependent inhibitory effect on the alveolar macrophages certainly represents a great advantage in terms of blocking the potentially lethal cytokine storm following a SARS-CoV-2 infection. However, free Spike protein, as well as circulating cells expressing the Spike protein, can generate additional effects whose consequences should be taken into due consideration. For instance, in vaccinees, Spike-protein-dependent unresponsiveness may occur in ACE-2-expressing macrophages other than the alveolar ones. Macrophages play a pivotal role in both innate and adaptive immunity. Hence, defects in the mechanisms underlying protection against both the adventitious and resident pathogens as well as emerging tumor cells may occur. In this scenario, macrophages with disabled immune functions may represent a relevant pathogenetic factor. Meanwhile, free and, possibly, cell-associated Spike proteins can interact with endothelial cells [56,57] thereby inducing cell damage, apoptosis, and inflammation. Unwanted consequences depend on the involved tissue compartment, with pericarditis, myocarditis, and hyper-coagulation being the most relevant adverse events.

Detailed and systematic investigations deciphering the biologic responses to anti-SARS-CoV-2 mRNA vaccines in all associated tissues would help in predicting and counteracting vaccine-induced side effects.

## Data Availability

Not applicable.
